# Optimization of Catheter Based rtPA Thrombolysis in a Novel In Vitro Clot Model for Intracerebral Hemorrhage

**DOI:** 10.1155/2017/5472936

**Published:** 2017-03-26

**Authors:** Naureen Keric, Julia Masomi-Bornwasser, Hendrik Müller-Werkmeister, Sven Rainer Kantelhardt, Jochem König, Oliver Kempski, Alf Giese

**Affiliations:** ^1^Department of Neurosurgery, University Medical Center, Johannes Gutenberg University, Mainz, Germany; ^2^Institute of Medical Biostatistics, Epidemiology and Informatics (IMBEI), University Medical Center, Johannes Gutenberg University, Mainz, Germany; ^3^Institute for Neurosurgical Pathophysiology, University Medical Center, Johannes Gutenberg University, Mainz, Germany; ^4^OrthoCentrum Hamburg, Hansastr. 1-3, Hamburg, Germany

## Abstract

Hematoma lysis with recombinant tissue plasminogen activator (rtPA) has emerged as an alternative therapy for spontaneous intracerebral hemorrhage (ICH). Optimal dose and schedule are still unclear. The aim of this study was to create a reliable in vitro blood clot model for investigation of optimal drug dose and timing. An in vitro clot model was established, using 25 mL and 50 mL of human blood. Catheters were placed into the clots and three groups, using intraclot application of rtPA, placebo, and catheter alone, were analyzed. Dose-response relationship, repetition, and duration of rtPA treatment and its effectiveness in aged clots were investigated. A significant relative end weight difference was found in rtPA treated clots compared to catheter alone (*p* = 0.002) and placebo treated clots (*p* < 0.001). Dose-response analysis revealed 95% effective dose around 1 mg rtPA in 25 and 50 mL clots. Approximately 80% of relative clot lysis could be achieved after 15 min incubation. Lysis of aged clots was less effective. A new clot model for in vitro investigation was established. Our data suggest that current protocols for rtPA based ICH therapy may be optimized by using less rtPA at shorter incubation times.

## 1. Introduction

Spontaneous intracerebral hemorrhage occurs in 10–15% of all stroke patients and is still a major cause of stroke-related death and disability [1–3]. Optimal therapy is still controversial. Prospective studies and randomized trials have shown equally poor outcome for best medical treatment or open surgery for evacuation of the hematoma, whereas one study suggests that the latter might be beneficial for selected patients with lobar hematomas not deeper than 1 cm from brain surface and a GCS between 9 and 12 [4–8].

In consideration of these results the interest in minimal invasive procedures has grown. Stereotactic frame-based or image-guided frame-less catheter placement and rtPA lysis of ICH have shown to be safe and effective in volume reduction [9–12]. In the past 2 decades this therapy has emerged to a well-established fast and easy procedure in many neurosurgical units [13]. However rtPA dosing mainly has been based on clinical experience. No published data are available from (i) in vitro or in vivo studies systematically analyzing optimal timing of rtPA administration, (ii) optimal dosing of the drug, and (iii) duration of efficient treatment. Not surprisingly some authors reported adverse events, which might be dose dependent, such as occurrence of an rtPA induced delayed cytotoxic perifocal edema. This was observed in animal models and clinical settings [14–17], while some other studies did not confirm these findings [18, 19]. A trial on minimal invasive ICH lysis using an intralesional catheter is presently conducted [20].

In order to establish optimal dose and timing for rtPA therapy, however high numbers of repetitive investigations are necessary to achieve valid and significant results. Studies in large ICH animal models are very expensive. An obvious alternative is an in vitro ICH model, which allows a high number of repetitions under controlled conditions. We here present an easy and robust in vitro ICH model in which we investigated optimal timing and dosing of rtPA lysis [21, 22].

## 2. Material and Methods

### 2.1. Blood Clot Preparation

We collected blood from the cubital vein of healthy volunteers into 20 mL syringes (BD Discardit, Germany). In vitro blood clots were produced from 25 mL or 50 mL of human blood supplemented with 10 IE of thrombin (bovine plasma thrombin, Sigma, Germany, final concentration 10 IE/500 *μ*L) in a balloon tightly closed and incubated 1.5 h in an incubator at 37°C (Heraeus Instruments, Germany). The application of thrombin has been adapted from an intravascular clot model [23, 24]. Before treatment clots were weighed, the clot and serum fraction were separated carefully by a fine mesh and weighed individually and afterwards placed back into the balloon for treatment. After clot production the clots were randomized to the different treatment groups.

### 2.2. In Vitro ICH Model

**Figure 1:**
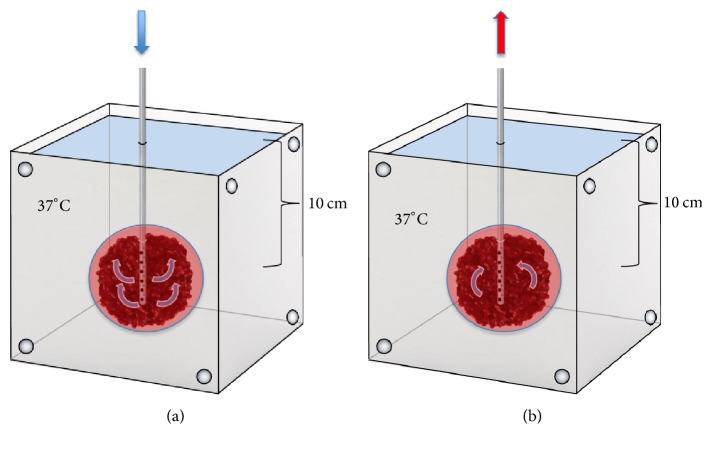
Blood clots submerged 10 cm in a 37°C water bath. An EVD is placed in all clots for drug administration (a). After treatment the liquefied fraction is drained by a gravity based drainage system (b).

An external ventricular drain (EVD) (Neuromedex® GmbH, Switzerland, 9 F, 30 cm length, 20 holes with 1 mm diameter) catheter was placed into all clots, mimicking the intracranial situation of a lysis catheter, and connected to a gravity based EVD drainage system (Neuromedex® GmbH, Switzerland) and placed 10 cm below the blood clot level. The clots were placed 10 cm below surface in a water bath at 37°C. Temperature was constantly monitored by a thermometer (PH Meter, WTW GmbH, Germany) (Figure 1).

After randomization and corresponding to the different experimental protocols the EVD system was opened and the liquefied fraction of the hematoma was drained by gravity. After treatment the remainder of each clot was weighed to assess the relative weight reduction of the clot.

### 2.3. Spontaneous Thrombolysis, Carrier Effect, and rtPA Lysis

In the first setting we investigated the amount of spontaneous lysis, a potential carrier effect, and the rtPA lysis effect. Group 1 (*n* = 6, 25 mL clots) was treated with an EVD to drain the liquid fraction after 1 h incubation in a 37°C water bath to quantify the spontaneous lysis process. In group 2 (*n* = 6, 25 mL clots) 5 mL of 0,9% NaCl was administered to the clots. Corresponding to group 1 the drain was opened after 1 h. Three clots (50 mL) were treated with a dose of 3 mg rtPA diluted in a volume of 5 mL in group 3. Drains were opened after 1 h incubation. Total volume of carrier (NaCl) or rtPA was 5 mL to exclude possible effects of different carrier volumes. Following this, relative clot weight reduction was compared for all groups.

### 2.4. Dose-Response Relationship

A dose-response relationship was evaluated in five groups each consisting of three 25 mL blood clots with five different doses of rtPA (0.5; 0.9; 1.2; 2; 3 mg, treatment time 60 min) using the clot model. Clots were weighed before and after treatment. Similar to this, 5 different doses of rtPA were applied in 50 mL clots (0.5; 0.9; 1.2; 2; 3; mg, treatment time 60 min). Furthermore each rtPA treated clot was compared to a placebo (5 mL 0.9% NaCl) treated clot of the same blood donor. The differences of weight of the treated and the control blood clots were statistically analyzed to assess the lysis effect of each rtPA dose.

### 2.5. Optimal Treatment Time

In order to investigate the optimal treatment time for rtPA, 25 mL clots were treated with an optimized rtPA dose of 1 mg by different periods of time. After rtPA administration, the EVD system was opened after 5, 15, 30, and 60 min (each time point in replicates of 3 clots). Relative weight reduction after treatment was compared.

### 2.6. Effectiveness of rtPA in Different Old Clots

Clots of different ages were produced as described above (1.5 h, 24 h, and 48 h; each group consisting of *n* = 3). One mg rtPA was applied repetitively four times. During each treatment rtPA was applied and remained in the clot for 15 min; then liquid fraction was drained for 10 min. Clots were weighed before and after treatment.

### 2.7. Statistical Analysis

We summarized results by reporting mean ± standard deviation. For comparison of spontaneous thrombolysis, carrier effect and rtPA lysis, and effectiveness of 1 mg rtPA in different aged clots statistical analysis was performed by one-way analysis of variance. 95% confidence intervals for all parameters were reported. Two-sided *p* values below 0.05 were considered as statistically significant. Analysis was performed with SigmaPlot 12.0. (Systat Software, Inc., USA) and GraphPad Prism (version 6.0).

For statistical analysis of the dose-response relationship of rtPA, we fitted a three-parameter logistic model and estimated the 50% and 95% effective dose with 95% confidence intervals based on the fitted model [25].

(1) $$\begin{array}{c} f\left(\;x,\left(\;b,d,e\;\right)\;\right)=\frac{d}{1+\exp\left\{b\left(\log\left(x\right)-\log\left(e\right)\right)\right\}}. \end{array}$$

Analysis was performed with R software, version 3.0.1: R Core Team (2013), R: A language and environment for statistical computing (R Foundation for Statistical Computing, Vienna, Austria, URL http://www.R-project.org/). For model fitting the R package DRC was used [25].

## 3. Results

### 3.1. Reliability of the Clot Model

**Figure 2:**
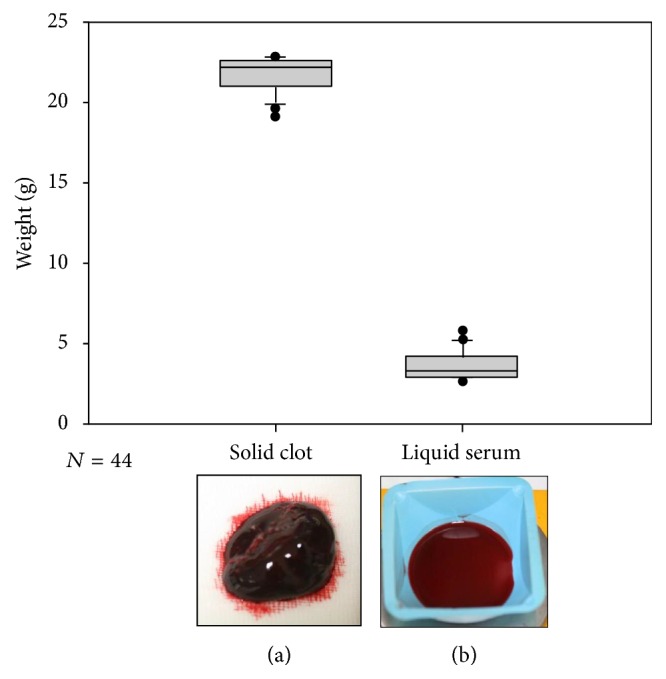
Clot formation in vitro; *n* = 44. Weight of clot (21.76 ± 1.03 g) and serum (3.81 ± 0.88 g) part is illustrated in box plots showing the low mean variance. (a) Showing the solid part of the blood clot and (b) the liquid serum part of the clot.

A total number of 44 clots of human blood were created. The solid clot and liquid serum parts were separated and weighed. The solid part had an average weight of 21.76 ± 1.03 g and the average liquid serum fraction was 3.81 ± 0.88 g. The low variance in weight shows the consistency of clot formation in this in vitro ICH model (Figures 2(a) and 2(b)) [21, 22].

### 3.2. Spontaneous Thrombolysis, Carrier Effect, and RtPA Lysis Effect

**Figure 3:**
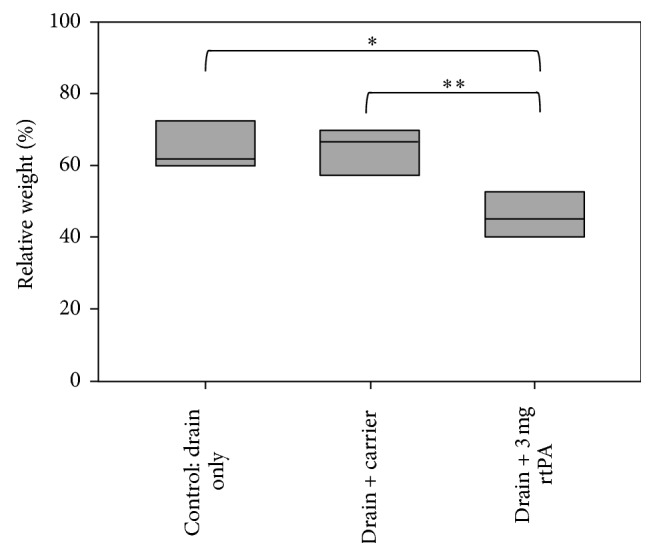
Comparison of spontaneous thrombolysis, carrier effect, and rtPA lysis as box plots (*n* = 6). The control group: blood clots treated with drain only had a relative weight of 64.96 ± 5.26%. Blood clots treated with 5 mL of carrier solution: 0.9% NaCl had a relative weight after treatment of 69.44 ± 6.67%. Clots treated with 3 mg of rtPA showed a relative weight of 46.01 ± 6.1%. ^*∗∗*^*p* < 0.001; ^*∗*^*p* = 0.002.

The control group (drain only) showed a mean relative end weight of 64.96 ± 5.26% of initial weight. The drain plus carrier treated clots with a mean relative end weight of 69.44 ± 6.67% showed no significant difference compared to the control group (*p* = 0.222). The rtPA (3 mg) treated clots with a relative end weight of 46.01  ±  6.1% showed a significant difference compared to control (*p* = 0.002) and to drain plus carrier alone treated clots (*p* < 0.001) (Figure 3) [21, 22].

### 3.3. Dose-Response Relationship of rtPA in 25 mL Clots

**Figure 4:**
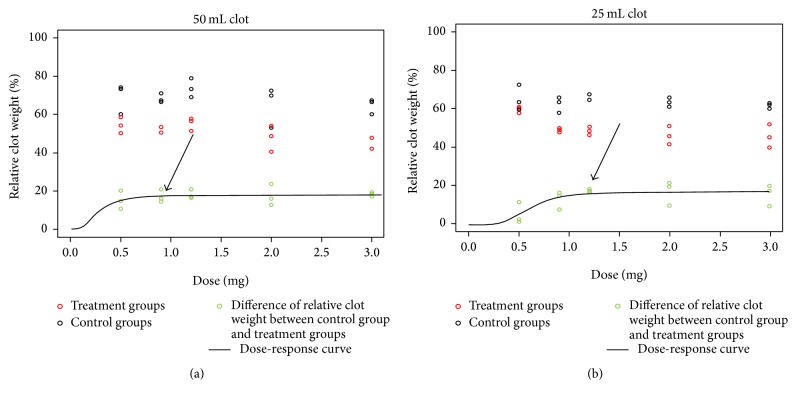
(a) A dose-response relationship was evaluated in five groups each consisting of three 25 mL blood clots with five different doses of rtPA (0.5; 0.9; 1.2; 2; 3 mg): treatment group (red) and control group (black). The relative posttreatment clot weight is shown on the *y*-axis. The differences of weight of the treated and the control blood clots showed the effect of lysis of each rtPA dose (green). The black line shows the dose-response relationship of rtPA. The arrow indicates the 95% effective dose of 1.2 ± 0.52 mg rtPA. (b) Similar to (a) a dose-response relationship was evaluated for 50 mL blood clots in five groups each consisting of three 50 mL blood clots with five different doses of rtPA (0.5; 0.9; 1.2; 2; 3 mg): treatment group (red) and control group (black). The relative posttreatment clot weight is shown on the *y*-axis. The differences of weight of the treated and the control blood clots showed the effect of lysis of each rtPA dose (green). The black line shows the dose-response relationship of rtPA. The arrow indicates the 95% effective rtPA dose of 0.84 ± 1.07 mg rtPA.

A dose-response relationship was evaluated in five groups each consisting of three 25 mL blood clots using five different doses of rtPA (0.5; 0.9; 1.2; 2; 3 mg) and compared to a placebo (5 mL 0,9% NaCl) treated clot from the same donor. Clots were weighed before and after treatment. The rtPA treated group showed a relative posttreatment weight of 60.03 ± 1.76% when treated with 0.5 mg rtPA, 49.06 ± 0.98% when treated with 0.9 mg rtPA, 48.78 ± 2.14% when treated with 1.2 mg rtPA, 46.34 ± 4.68% when treated with 2 mg rtPA, and 46.01 ± 6.1% when treated with 3 mg rtPA. The control group had a mean relative posttreatment weight of 64.04 ± 3.67%. The 95% effective dose (ED95) was 1.2 ± 0.52 mg rtPA; the 50% effective dose (ED50) was 0.6 ± 0.1 mg (Figure 4(a)) [21, 22].

### 3.4. Dose-Response Relationship of rtPA in 50 mL Clots

The same experiment was performed in 50 mL clots. The treatment group showed a relative posttreatment weight of 54.23 ± 4.41% treated with 0.5 mg rtPA, 51.41 ± 1.79% treated with 0.9 mg rtPA, 55.23 ± 3.36% treated with 1.2 mg rtPA, 47.91 ± 6.96% treated with 2 mg rtPA, and 45.98 ± 3.06% treated with 3 mg rtPA. The control group had a mean relative posttreatment weight of 68.94 ± 6.52%. The 95% effective dose (ED95) was 0.84 ± 1.07 mg rtPA; the 50% effective dose (ED50) was 0.28 ± 0.35 mg (Figure 4(b)) [21, 22].

### 3.5. Optimal Treatment Time and Lysis Rate

**Figure 5:**
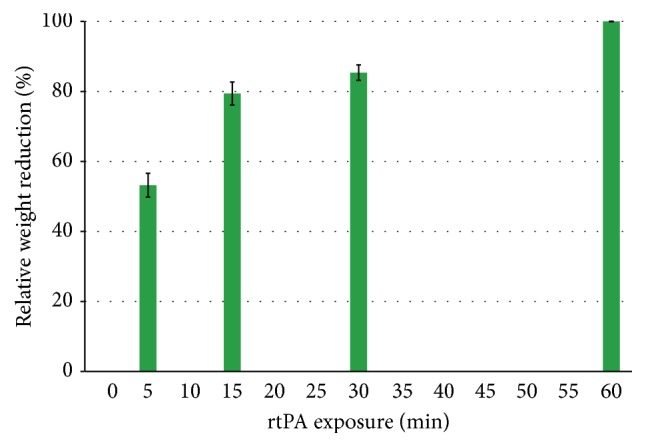
The graph shows normalized relative end weights after different time exposures of the blood clots to rtPA for 5, 15, 30, and 60 min determining that 100% lysis takes place within 60 min. Error bars represent standard error per condition; values are rescaled for 100% per 60 min; app. 80% lysis happens during 15 min. Each bar represents *n* = 3 clots.

Assuming a maximum lysis rate of 100% after 1 h, lysis rates after different exposure times to rtPA were analyzed in 25 mL clots. The normalized rate of lysis after 5 min exposure to rtPA was 53.22 ± 3.9%; after 15 min it was 79.41 ± 1.7% and after 30 min 85.38 ± 1.5% (Figure 5) [21, 22].

### 3.6. Effectiveness of rtPA in Clots of Different Age

**Figure 6:**
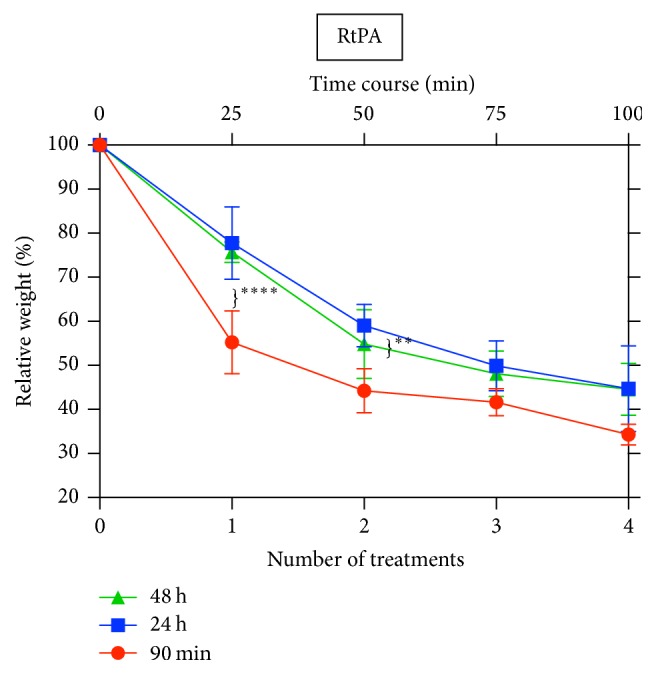
Effectiveness of 1 mg rtPA in different old clots: 90 min (red), 24 h (blue), and 48 h (green): the graph shows the relative weight in percent (*y*-axis) after 1 till 4 treatment cycles (*x*-axis) (*n* = 3). On top axis the time course shows cycles of 25 min, consisting of 15 min of rtPA-exposure and 10 min drainage period. *∗∗∗∗* indicates the significant weight difference of 90 min old clots compared to 24 h (*p* < 0.0001) and 48 h old clots (*p* = 0.0002) during the first treatment. *∗∗* indicates the significant weight difference of the 90 min old clots compared to 24 h old clots (*p* = 0.0059) during the second treatment.

Fibrinolytic treatment with rtPA is less effective in aged clots. There was a significant weight difference of 90 min old clots compared to 24 (*p* < 0.0001) and 48 h old clots (*p* = 0.0002) during the first treatment (Table 1). During the second treatment there was still a significant difference between the 90 min aged clots compared to 24 h aged clots (*p* = 0.0059) (Table 1). Repetitive rtPA treatment showed decreasing effectiveness in weight reduction. The bulk weight reduction was achieved by the first two treatments (Figure 6) [21, 22].

## 4. Discussion

In the present study to the best of our knowledge we investigated for the first time a systematic analysis of fibrinolytic therapy of rtPA (supplemental flowchart illustrates the experimental work; see Supplementary Material available online at https://doi.org/10.1155/2017/5472936). We established a novel in vitro ICH clot model [21, 22]. 44 blood clots were produced from 25 or 50 mL of human blood from healthy volunteers. The supplementary use of thrombin according to published methods of in vitro micro intravascular clots stabilized the clot independent from donor [23, 24]. The model proved to be highly reproducible in terms of clot formation of solid hematomas and serum fraction (Figure 2) [21, 22]. This setting allowed an easy workflow and a high number of repetitions under controlled conditions and avoided the need of a large ICH animal model [26].

In the first experimental series we evaluated the effect of rtPA lysis in comparison to control groups. Our findings indicate that normal saline irrigation of clots has no significant fibrinolytic effect [21, 22]. The rtPA treated clots in contrast had a significant loss of their relative end weight, confirming that fibrinolysis takes place in this experimental in vitro setting (Figure 3). These results demonstrating an app. 55% volume reduction after a single dose of 3 mg rtPA are in line with several experimental and clinical studies investigating the fibrinolytic potential of rtPA in ICH [9–19, 26–28]. In one of the first clinical series reported already 2 decades ago Lippitz et al. reported on fibrinolytic therapy after initial stereotactic aspiration of the hematoma. In 10 patients the authors yielded about 60% volume reduction by aspiration and a total of 84% hematoma removal after additional repetitive rtPA administration of 3 mg daily over 1–3 days [9]. The latest published study, an analysis of a Phase II trial patient collective for minimal invasive surgery with rtPA, showed that this therapy is well tolerated and effective. The authors even found a decrease of perihematomal edema in the rtPA and aspiration treated group compared to the aspiration only treated group. They assume no neurotoxic rtPA effects on perifocal brain tissue when it is administered into the clot. Moreover the larger hematoma volume and reduction of toxic metabolites in the aspiration and rtPA group may lead to a decreased perifocal edema [19]. Until now no clear correlation of rtPA doses and the occurrence or size of a perifocal edema has been published. Interestingly the animal and clinical studies reporting of rtPA related perifocal edema applied relative high cumulative doses of rtPA [14, 16, 17, 26]. The ongoing phase III MISTIE trial will reveal possible dose-related side effects.

To further characterize the optimal dose of rtPA in different clot sizes, we performed a dose-response analysis in 25 mL and 50 mL blood clots. The 95% effective dose of rtPA in 25 mL clots was 1.2 ± 0.52 mg and 0.84 ± 1.07 mg in 50 mL clots [21, 22]. We interpreted the lower dose in larger clot as not significant. In the Phase II MISTIE trial the rtPA dose was evaluated in 2 arms, while arm 1 received 0.3 mg every 8 hours, up to 9 times; arm 2 received 1 mg every 8 hours and up to 9 times in 72 hours. The investigators chose these dose regimens with reference to the recommendation of the American Heart Association in the application of intravenous rtPA and by the experiences of different clinical centers [9–14]. Some authors applied 1 mg rtPA per 10 mL hematoma, whereas hematoma volume was assessed by the formula *A*  ×  *B*  ×  *C*/2. The total rtPA doses ranged in these series depending on hematoma volume from 5 to 16 mg [10, 14, 15]. In our in vitro study surprisingly a larger clot did not require a higher rtPA dose. This might result from the relative hematoma surface, which can be reached by rtPA molecules per administration via the EVD. Possibly the catheter perforations and design play a role in this phenomenon. However the relative activity of rtPA in 1 mg rtPA seems to be sufficient or excessive even for larger hematoma volumes like 50 mL [21, 22]. The effect of repetition and timing remain unclear.

After assessment of an optimal rtPA dose we investigated the optimal exposure time of the clot to rtPA. In the published clinical series already mentioned above and in the protocol of the phase III MISTIE trial the drain was closed for 1 hour after rtPA application. Then the drain was opened for passive flow by gravity [9–11, 14, 15, 18–20]. Considering this clinical practice we assumed that the fibrinolytic effect, which can be reached by a single rtPA dose in 1 hour, was determined as 100%. Assuming this, in our in vitro series the lysis rates after 5 min exposure to rtPA were 53.22 ± 3.9%, after 15 min 79.41 ± 1.7%, and after 30 min 85.38 ± 1.5% [21, 22]. These results correspond well to the half-life of rtPA, which is about 6 min [29]. But it raises the question, whether it is necessary to close drains for 1 hour, if app. 80% of the lysis can be achieved within 15 min [21, 22]. Translating these findings to the clinical situation with a faster opening of the drain could lower time of increased intracranial pressure and increase the effectiveness of hematoma evacuation.

The question of rtPA efficacy in older hematomas is still a matter of debate. The phase III MISTIE protocol excludes patients with symptoms more than 24 h prior to the initial diagnostic CT scan and surgery should be intended in 72 hours after ictus [20]. In our in vitro series we found a significant higher lysis rate in newly formed clots of 1.5 h compared to the 24 h and 48 h old clots [21, 22]. There was no difference in the lysis rate of 24 h and 48 old clots, suggesting that the relevant changes causing rtPA resistance take place within the first 24 h. Furthermore, repetitive rtPA administrations did not result in a linear decrease of clot volume. The largest volume reduction occurred after the first 2 rtPA applications.

## 5. Limitation and Advantages of This Model

These results, however, have to be interpreted with caution. This in vitro model does not consider the perifocal environment of the brain tissue surrounding the intracerebral hemorrhage. Many inhibiting and activating factors may influence the maturation of clots but also the activity of the administered drugs. Large animal models may be superior to our in vitro model in assessing this question.

The advantages of this model are its reproducibility and reliability of clot size and structure and the usefulness in numerous future experiments, focusing not only on rtPA kinetics. Effects and kinetics of several other lytic drugs and their combination may be rapidly compared and investigated in this model. Furthermore the lytic activity of different ultrasound modes and physics can be assessed easily in this in vitro model. The model offers the perspective of assessing an individualized fibrinolytic therapy using lytic drugs alone, sonothrombolysis alone, or a combination of lytic drugs and sonothrombolysis. Therapeutic issues concerning clot age and coagulation status are important, which can be easily screened in such a model system before testing in an animal model and finally in the patient.

## 6. Conclusion

We established an easy and robust in vitro model of ICH, which allows a high number of repetitive experiments under controlled conditions. We applied this model to assess the optimal dose and timing of rtPA lysis in human blood clots and found a surprisingly low optimal dose of only 1 mg rtPA independent of the clot size (25, 50 mL, resp.). Further we showed that 80% of the lysis occurs within the first 15 min of incubation. The data suggests that current protocols for rtPA based ICH therapy could possibly be optimized by using smaller doses and shorter incubation times. This might in the future allow a faster reduction of intracranial pressure than achieved in current clinical protocols.

## Supplementary Material

This flowchart illustrates the experimental workflow of establishing an in vitro clot model and the further stepwise investigations and their key results.

## Figures and Tables

**Table 1:** Relative weight after repetitive treatment with 1 mg rtPA in different aged clots: 90 min, 24 h, and 48 h.

Treatment cycles	90 min	24 h	48 h	Statistics
1	55.23 ± 7.13%	77.78 ± 8.2%	75.71 ± 2.35%	90 min versus 24 h: *p* < 0.0001 90 min versus 48 h: *p* = 0.0002
2	44.23 ± 5.04%	59.02 ± 4.78%	54.83 ± 7.83%	90 min versus 24 h: *p* = 0.0059
3	41.62 ± 3.11%	49.89 ± 5.65%	48.07 ± 5.17%	
4	34.28 ± 2.33%	44.69 ± 9.77%	44.54 ± 5.86%	
