# Supplementary material for: Optimization of Catheter Based rtPA Thrombolysis in a Novel In Vitro Clot Model for Intracerebral Hemorrhage
Source: Biomed Res Int. 2017 Mar 26;2017:5472936. doi: 10.1155/2017/5472936 (PMC5385248; doi:10.1155/2017/5472936)
Supplement: Supplementary file 1 — This flowchart illustrates the experimental workflow of establishing an in vitro clot model and the further stepwise investigations and their key results. [file 5472936.f1.pdf]

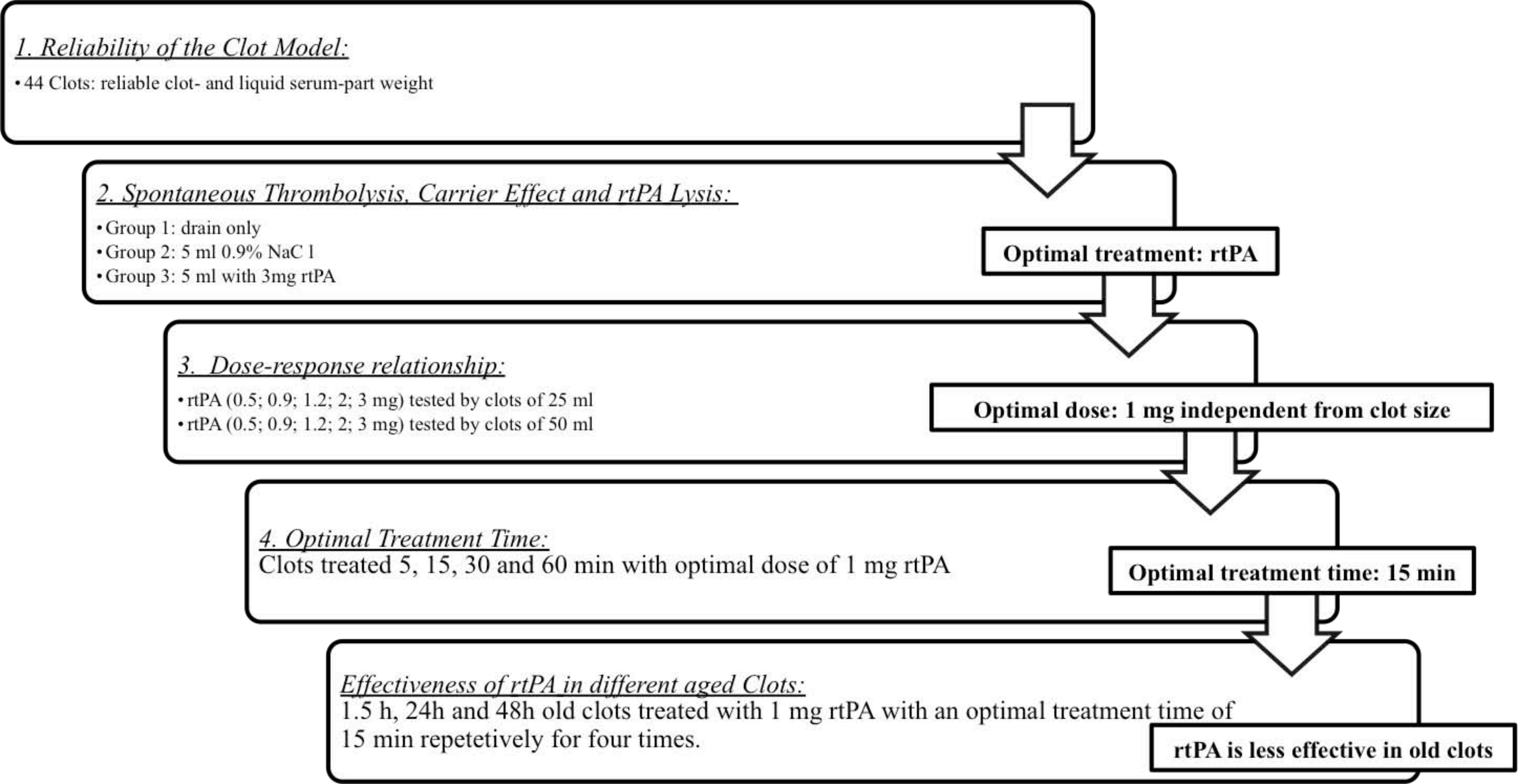
1. Reliability of the Clot Model:
• 44 Clots: reliable clot- and liquid serum-part weight
2. Spontaneous Thrombolysis, Carrier Effect and rtPA Lysis:
• Group 1: drain only
• Group 2: 5 ml 0.9% NaC l
• Group 3: 5 ml with 3mg rtPA
Optimal treatment: rtPA
3. Dose-response relationship:
• rtPA (0.5; 0.9; 1.2; 2; 3 mg) tested by clots of 25 ml
• rtPA (0.5; 0.9; 1.2; 2; 3 mg) tested by clots of 50 ml
Optimal dose: 1 mg independent from clot size
4. Optimal Treatment Time:
Clots treated 5, 15, 30 and 60 min with optimal dose of 1 mg rtPA
Optimal treatment time: 15 min
Effectiveness of rtPA in different aged Clots:
1.5 h, 24h and 48h old clots treated with 1 mg rtPA with an optimal treatment time of 15 min repetetively for four times.
rtPA is less effective in old clots
